# Demand- and supply-side determinants of diphtheria-pertussis-tetanus nonvaccination and dropout in rural India

**DOI:** 10.1016/j.vaccine.2016.12.024

**Published:** 2017-02-15

**Authors:** Arpita Ghosh, Ramanan Laxminarayan

**Affiliations:** a Public Health Foundation of India, Gurgaon, Haryana, India; b Center for Disease Dynamics, Economics & Policy, Washington, DC, USA; c Princeton University, Princeton, NJ, USA

**Keywords:** ANM, auxiliary nurse midwife, ASHA, accredited social health activist, CES, Coverage Evaluation Survey, DLHS, District Level Household and Facility Survey, DPT, diphtheria-pertussis-tetanus, OPV, oral polio vaccine, PHC, primary health centre, UNICEF, United Nations Children’s Fund, Vaccine uptake, Partial or incomplete immunization, Multilevel modeling, District Level Household and Facility Survey (DLHS-3) 2007–08

## Abstract

**Background:**

Although 93% of 12- to 23-month-old children in India receive at least one vaccine, typically Bacillus Calmette–Guérin, only 75% complete the recommended three doses of diphtheria-pertussis-tetanus (DPT, also referred to as DTP) vaccine. Determinants can be different for nonvaccination and dropout but have not been examined in earlier studies. We use the three-dose DPT series as a proxy for the full sequence of recommended childhood vaccines and examine the determinants of DPT nonvaccination and dropout between doses 1 and 3.

**Methods:**

We analyzed data on 75,728 6- to 23-month-old children in villages across India to study demand- and supply-side factors determining nonvaccination with DPT and dropout between DPT doses 1 and 3, using a multilevel approach. Data come from the District Level Household and Facility Survey 3 (2007–08).

**Results:**

Individual- and household-level factors were associated with both DPT nonvaccination and dropout between doses 1 and 3. Children whose mothers had no schooling were 2.3 times more likely not to receive any DPT vaccination and 1.5 times more likely to drop out between DPT doses 1 and 3, compared with children whose mothers had 10 or more years of schooling. Although supply-side factors related to availability of public health facilities and immunization-related health workers in villages were not correlated with dropout between DPT doses 1 and 3, children in districts where 46% or more villages had a healthcare subcentre were 1.5 times more likely to receive at least one dose of DPT vaccine compared with children in districts where 30% or fewer villages had subcentres.

**Conclusions:**

Nonvaccination with DPT in India is influenced by village- and district-level contextual factors over and above individuals’ background characteristics. Dropout between DPT doses 1 and 3 is associated more strongly with demand-side factors than with village- and district-level supply-side factors.

## 1 Introduction

Coverage with the third dose of diphtheria-pertussis-tetanus vaccine (DPT3) is a widely used indicator of the performance of countries’ routine immunization services [1], [2]. In 2014, Indian children accounted for 22% of the 18.7 million children worldwide who had not received three doses of DPT by age one [3]. Low DPT3 coverage is caused by a combination of low uptakes of dose 1 and high dropout rates among infants who receive the first dose but not the second or third. During 2014, nearly 2.5 million children in India did not receive a single DPT dose, while more than 1.5 million received only one or two DPT doses [3]. Our hypothesis is that the barriers to immunization for children who have never received a single dose of DPT may be different from those for children who have received one or two doses but not completed the full series.

Studies conducted worldwide and in India have documented the determinants of vaccination coverage and have catalogued the strategies that have proven effective in improving immunization coverage [4], [5], [6], [7]. Supply-side factors include availability and access to healthcare facilities, infrastructure, staffing, vaccine and service delivery management, budget allocation, and knowledge of the workers who administer vaccines [8], [9], [10], [11], [12]. On the demand side, the factors associated with vaccination coverage in children typically include child’s birth order and sex, parents’ level of education, their employment status and type of occupation, immunization-related beliefs, mother’s general health knowledge and awareness, health-seeking behavior, caste, religion, and household wealth index [10], [11], [12], [13], [14], [15], [16], [17], [18], [19], [20], [21], [22], [23], [24], [25], [26], [27]. Rammohan et al. in their ecological analysis show that district-level differences in immunization coverage are correlated with district-level per capita income and maternal education [28]. Studies using a multilevel approach revealed associations with contextual factors, such as urban place of residence, countries with high fertility rates, communities with high illiteracy rates, municipalities with religious objection to vaccination, communities with fewer deliveries attended by health personnel, and postcode areas with lower socio-economic status [15], [17], [25], [29], [30], [31]

Risk factors for incomplete vaccination may be different from the risk factors for nonvaccination. A global review of the grey literature by Favin et al. found that the main reasons for incomplete vaccination were bad experiences at the immunization centre (health workers’ poor treatment of mothers, long waiting time, unavailability of drugs), missed opportunities (health workers’ refusal to immunize sick children, turning away a child who lacked a vaccination card), fear of side effects, and not knowing whether to return or when. Rainey et al. in their systematic review noted that factors such as parental education, cultural mores, and religious beliefs were more likely to be associated with nonvaccination than with incomplete vaccination [32], [33].

A handful of studies have investigated reasons behind dropout after the first dose of DPT or oral polio vaccine (OPV), albeit within a very limited scope. Usman et al. in their cohort study involving 366 mother-infant pairs from six rural immunization centres around Karachi, Pakistan, found that children who received DPT dose 1 in a timely manner and lived closer to the immunization site were more likely to receive the subsequent doses [34]. A prospective cohort study in 21 health facilities in Ibadan North, Nigeria, found that the type of health facility attended was the only significant factor for completion of DPT3 among infants who received the first dose of OPV and/or DPT [35]. Randomized controlled trials in Pakistan demonstrated that providing mothers with a redesigned immunization card and home- or centre-based education on the importance of vaccines help improve DPT3 completion rates [36], [37], [38]. Predictors of nonvaccination with DPT and dropout between doses 1 and 3 have not been systematically studied in India. We sought to address this gap by analyzing vaccination data on 6- to 23-month-old Indian children from a nationally representative survey.

## 2 Methods

### 2.1 Data

We use data from the third round of the District Level Household and Facility Survey (DLHS-3), conducted during 2007–08. In this survey, 643,944 ever-married women aged 15–49 years from 720,320 households in 34 Indian states and union territories were interviewed on reproductive services and child health. Receipt of vaccination doses was recorded from children’s vaccination cards shown to the enumerator. If a card was not available, the mother was asked about the vaccines the child had received. Village questionnaires yielded information on availability of health, education, and other facilities in villages, and the facility-level survey gathered information on human resources, infrastructure, and services in the health facilities. Details on the survey methodology, sampling design, and the questionnaires are available elsewhere [39] and described briefly in the Appendix.

### 2.2 Variables

Combining data from the immunization cards and maternal recall, we defined three DPT vaccination outcomes: receipt of at least one dose of DPT (denoted by DPT1), receipt of all three DPT doses (denoted by DPT3), and receipt of all three DPT doses versus receipt of only one or two DPT doses (denoted by DPT3|DPT1). Typically, studies have modeled either nonvaccination with DPT or receipt of all three DPT doses. That P(DPT3) can be modeled as P(DPT1)P(DPT3|DPT1), which essentially means that receipt of at least one DPT dose and receipt of all three doses among infants who received at least one DPT dose are events contributing to and culminating in the completion of the three-dose DPT series, is not effectively used in standard analyses. Factors that affect nonvaccination with DPT, or 1-P(DPT1), may be different from factors that affect dropout from DPT vaccination, or 1-P(DPT3|DPT1). We examine the probability of receiving all three DPT doses among infants who had received at least one DPT dose, or P(DPT3|DPT1), to directly examine the risk factors for dropout between doses 1 and 3.

We included individual- and household-level risk factors such as characteristics of the child, mother, and the household: the child’s sex, birth order, and their interaction; the mother’s and her partner’s years of schooling; her knowledge of diarrhoea management, awareness of immunization-related messages and colostrum feeding practices; religion and caste of the head of the household; and household wealth quintile. In addition, we considered indicators of village-level infrastructure, such as availability of electricity; availability of a subcentre and primary health centre (PHC) in the village and all-weather road connectivity with the subcentre or PHC; and availability in villages of auxiliary nurse midwives (ANMs) and accredited social health activists (ASHAs)—community health workers who help mobilize and vaccinate the rural population in India. We also included variables summarizing the district’s demographic and socioeconomic profile and the health infrastructure: percentages of fourth or higher birth order children, women with six or more years of schooling, households belonging to the richest wealth quintile, and villages with a subcentre. We considered a state-level dummy variable indicating the nine ‘high-focus’ states with poor infrastructure and low public health indicators. Further details on variables are provided in the Appendix.

### 2.3 Statistical analyses

All analyses were confined to the 75,728 youngest living children, 6–23 months old, from villages across India, except union territories and Goa. We considered children 6 months or older so that all children have had opportunity to receive all three recommended DPT doses and also children less than two years old so that mothers would be able to recollect their vaccination history. For studying the effects of village-level structural determinants on vaccine uptake, we restricted the analyses to children from rural areas only and therefore excluded union territories and Goa, which have very few villages. Coverage for DPT1 and DPT3 was calculated by dividing the number of children receiving at least one DPT dose and all three DPT doses, respectively, by the total number of children aged 6–23 months. Coverage for DPT3|DPT1 was calculated by dividing the number of children receiving all three DPT doses by the number of children who received at least one dose of DPT. Estimates of prevalence and coverage rates were calculated using survey weights to account for unequal selection probabilities, and standard errors were adjusted for clustering at the village level. We performed multivariate logistic regressions to identify determinants of nonvaccination with DPT and dropout between doses 1 and 3. To account for the hierarchical nature of the data, we used multilevel logistic regression (see Appendix for technical details) to model the probability of the two binary outcomes—receipt of at least one dose of DPT and receipt of all three DPT doses among infants who received at least one dose of DPT. We conducted the descriptive analyses in R v.3.2.4 and used *MLwiN* (version 2.32) for fitting the multilevel models.

## 3 Results

The study sample consists of 75,728 children 6–23 months old from villages across India, except for union territories and Goa. Characteristics of the study sample are summarized in Table 1. Nearly 50% of the mothers have never attended any school, and 44% of the mothers have no knowledge of actions to take if a child has diarrhoea. However, 94% of mothers report that they have seen, heard, or read immunization-related messages. We find that 73% of the children have vaccination cards, though only 44% of the mothers were able to show the cards to the enumerator. Although only 40% and 14% of the villages have subcentres and PHCs, respectively, 84% of the villages are connected by road to a subcentre or PHC.

**Fig. 1:**
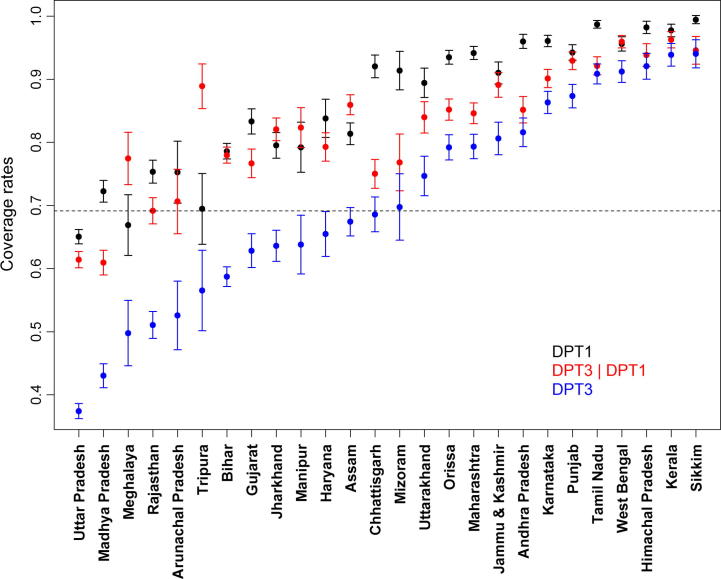
Coverage rates for three outcomes: (1) receipt of at least one dose of DPT (DPT1), (2) receipt of all three DPT doses among infants who received at least one dose of DPT (DPT3|DPT1), and (3) completion of three-dose series (DPT3), across rural parts of 26 states in India. The dots represent estimates of coverage rates, and the horizontal bars around the dots represent 95% confidence limits. Estimates and standard errors use survey weights and account for clustering at the primary sampling unit level. The states are arranged in ascending order of DPT3 coverage, and the dotted line indicates median DPT3 coverage of 69% across states.

Fig. 1 presents coverage for receipt of at least one DPT dose (i.e., DPT1) and receipt of all three DPT doses among infants who received at least one dose of DPT (i.e., DPT3|DPT1), in addition to the often-examined DPT3 coverage, across 26 major states, by ascending DPT3 coverage. We observe substantial variation in DPT3 coverage, from 39% in Uttar Pradesh to 94% in Sikkim. DPT3 coverage rates alone, however, do not help explain the relative contribution of DPT nonvaccination versus DPT1–3 dropout to the issue of low DPT3 coverage. For example, Bihar and Madhya Pradesh have similar DPT1 coverage rates, but the DPT3|DPT1 coverage rate is higher in Bihar, resulting in higher DPT3 coverage. Chhattisgarh starts with DPT1 coverage as high as 92% but ends up having only 71% DPT3 coverage.

Next, we examine the coverage rates for different DPT vaccination outcomes among infants, by background characteristics (Table 2). The last column, denoting DPT3 coverage, shows wide variation in coverage rates across different levels of various socioeconomic and demographic determinants. For example, DPT3 coverage is 49% for children born to mothers without any schooling but 85% for children whose mothers have grade 10 or higher education. DPT1 coverage rates also vary considerably between different subgroups of children—from 50% for children whose mothers have not seen, heard, or read immunization-related messages to 96% for children of mothers with grade 10 or higher education. The wide variation in DPT3 coverage rates is largely explained by the variation in DPT1 coverage rates across different strata. Coverage rates for DPT3|DPT1 are again rather variable (69–89%), albeit less than DPT1.

Lastly, we examine how individual- and household-level socioeconomic and demographic characteristics, together with village- and district-level variables, differentially affect nonvaccination with DPT and dropout between doses 1 and 3 (Table 3). We note that the individual-level demand-side factors—child’s birth order, mother’s education, her general health knowledge and practices, her immunization-related knowledge, caste and religion of household head, and household wealth quintile—affect both nonvaccination and dropout between doses 1 and 3. We note, however, a considerable drop in the effect size estimates for receipt of all three doses among infants who have received at least one dose of DPT, for the majority of these individual- and household-level risk factors.

Covariates related to the availability of immunization-related workers and public health facilities in the village are not found to be correlated with the completion of the three-dose DPT series among infants who have received at least one DPT dose. However, for receipt of at least one dose of DPT, availability of ASHAs and ANMs in the village is critical. Indicators of village-level general infrastructure, like availability of electricity and all-weather road connectivity with the subcentre or PHC, are associated with both higher chances of receipt of at least one DPT dose and higher chances of completing the three-dose series among infants who have received at least one dose of DPT. We find a strong association between DPT outcomes and the percentage of higher birth order children in the district. The district-level percentage of villages with subcentres is correlated with receipt of at least one dose of DPT but not with completion of the DPT series among those who have received at least one DPT dose.

## 4 Discussion

Our study considers both supply- and demand-side determinants of immunization and allows us to evaluate their relative importance for both DPT outcomes—nonvaccination and dropout between doses 1 and 3. Our multilevel analytical framework helps incorporate the hierarchical structure in the data and, more importantly, study the variation in child’s DPT vaccination status in terms of both individual-level and district- and village-level characteristics.

We uncovered important differences in the epidemiology of nonreceipt of and dropout from DPT vaccination. States having more than 90% DPT1 coverage have dropout rates varying between 4% (West Bengal) and 23% (Chhattisgarh). High dropout rates coupled with high DPT1 coverage suggest that many mothers who visit a health facility for dose 1 but do not return for the subsequent ones may be dissatisfied with the services or not made aware that three doses are needed. Studies in other countries have found that designing reminder-type immunization cards [38], sending text message reminders to parents [40], and improving maternal knowledge of vaccines [36], [38] are effective strategies in reducing vaccination dropout rates.

Consistent with earlier findings [16], [26], [41], we found father’s education to have a strong and independent effect on DPT uptake. Effect size estimates for the individual- and household-level factors, although found significant for both DPT outcomes, were smaller for DPT 1–3 dropout, particularly for mothers’ lack of awareness of immunization-related messages (odds ratio was 4.2 for nonvaccination with DPT compared with 1.4 for DPT1–3 dropout). This fits with the findings, albeit for the full sequence of recommended childhood vaccines, from the Coverage Evaluation Survey, a nationwide survey undertaken by the United Nations Children’s Fund (UNICEF) in India in 2009 [42]: higher percentages of mothers of nonvaccinated children mentioned ‘did not feel the need for immunization’, ‘did not know what vaccines are needed and when’, and ‘did not know where to take child for immunization’ as reasons than did mothers with partially vaccinated children (28%, 31%, and 16% versus 25%, 26%, and 10%, respectively).

The percentage of fourth or higher birth order children in the district, a proxy for district-level fertility rate, was strongly associated with both DPT outcomes, in line with the finding from a multilevel study in sub-Saharan Africa that children in countries with high fertility rates are less likely to receive all three DPT doses [29]. However, the district-level percentage of women with primary education, in spite of an impressive line-up of evidence on effect of the educational context on children’s immunization [14], [29], [43], was not correlated with DPT1 and correlated only marginally with DPT3|DPT1. Earlier studies have examined the role of vaccination cards for the uptake of vaccinations against DPT, polio, and measles [10], [37], [38]. Among the infants who received at least one dose of DPT, we compared mothers having no vaccination cards with mothers reporting having vaccination cards but unable to show them to the enumerator. We observed that children whose mothers reported that the child possessed a vaccination card were 1.4 times more likely to have received dose 3, confirming findings from previous research.

Our study has important limitations. We studied the effect of availability of public health facilities and health workers in villages but were unable to explain the effect of quality of services at these facilities—availability of vaccines and equipment, staffing, knowledge of the workers who perform vaccination, and infrastructure—on vaccination outcomes. Earlier studies have demonstrated that facilities with staffing and infrastructure problems and poor quality of services promote dissatisfaction and decrease demand [9], [11], [44]. In DLHS-3, data from the facility-level survey could not be reliably linked to information on children’s immunization, even at the village level. We also failed to account for variables such as women’s decision-making ability, which has been shown to have a positive effect on a mother’s health-seeking behavior and therefore child health outcomes [14], [42], [45], [46]. Unfortunately, DLHS-3 does not have data on mothers’ decision-making.

For the vaccination status of children for whom the enumerator was not able to see the vaccination card, we relied on the mothers’ recall. The accuracy of a mother’s recall of her child’s vaccinations could not be assessed because mothers’ responses were sought only in cases where vaccination cards were not available. Moreover, we treated the DPT outcomes as binary variables by collapsing categories of DPT doses, which may lead to loss of information. Instead, we can retain the ordinal nature of the number of DPT doses variable and fit a random intercepts cumulative logit model, under the proportional odds assumption. Finally, because DLHS-3 was a cross-sectional survey, we could study only associations between risk factors and DPT outcomes and were not able to make causal inferences.

## 5 Conclusions

Although immunization strategies in developing countries have often focused on ensuring a reliable supply of health services [47], [48], [49], [50], [51], we found no evidence that availability of health facilities and community health workers who mobilize and vaccinate the rural population improved the likelihood that a child with DPT1 would complete DPT3. The oft-quoted maternal and household characteristics that are important predictors of full immunization were significantly associated with dropout between DPT doses 1 and 3. Therefore, policy makers aiming to improve DPT completion rates may consider demand-side approaches, such as giving parents information on immunization, providing health education, and partnering with and engaging communities, which would also help tackle the broader social inequity issue.

## Conflict of interest

None of the authors have any competing interests.

## Authors’ contributions

RL conceived of the study question. AG conducted the statistical analyses, interpreted the data, and drafted the manuscript. RL contributed to interpretation of data, reviewed the manuscript, and helped finalize the draft. All authors approved the version submitted for publication.

## Funding

AG was supported by a Wellcome Trust Capacity Strengthening Strategic Award to the Public Health Foundation of India and a consortium of UK universities (WT084754/Z/08/A). This project was supported by a grant by the Bill & Melinda Gates Foundation to the Immunization Technical Support Unit at the Public Health Foundation of India.

## Figures and Tables

**Table 1:** Distribution of study sample of 75,728 infants aged 6–23 months, in villages across India, by background characteristics.

**Individual- and household-level characteristics**	**N**	**Percentage**
**Sex, birth order (n** **=** **75,701)**		
Boy, birth order ⩽3	30,049	40.0
Girl, birth order ⩽3	27,117	36.0
Boy, birth order ⩾4	9724	12.6
Girl, birth order ⩾4	8811	11.4

**Child’s vaccination card (n** **=** **75,724)**		
Card is seen	33,255	43.9
Child has card but not seen	21,986	29.2
No card	20,483	26.9

**Mother’s education (n** **=** **75,702)**		
0 years of schooling	37,512	48.7
Years of schooling: 1–5	11,786	15.7
Years of schooling: 6–9	15,792	21.3
Years of schooling: 10 or more	10,612	14.3

**Partner’s education (n** **=** **75,083)**		
0 years of schooling	20,259	26.6
Years of schooling: 1–5	12,838	17.1
Years of schooling: 6–9	20,907	28.0
Years of schooling: 10 or more	21,079	28.3

**Mother’s knowledge about diarrhoea management, on scale of 0–5 (n** **=** **75,661)**		
0	33,457	44.1
1	21,689	28.6
2	15,165	20.1
3–5	5350	7.2
**Mother fed colostrum during last live birth (n** **=** **74,686)**	58,709	79.0
**Mother has seen, heard, or read messages related to immunization (n** **=** **75,727)**	70,637	93.6
**Household head is Muslim (n** **=** **75,723)**	9816	12.8
**Household head belongs to scheduled tribe (n** **=** **75,645)**	14,150	19.2

**Household wealth quintile (n** **=** **75,712)**		
Poorest	18,725	24.3
2nd quintile	18,742	24.5
3rd quintile	16,970	22.7
4th quintile	14,118	19.0
Richest	7157	9.6

**Village-level characteristics (n** **=** **20,433)**	**N**	**Percentage**

Does not have electricity	3518	17.2
Has subcentre	8162	40.2
Has PHC	2804	13.7
Is connected by all-weather road to subcentre or PHC	17,110	83.7
Has ANM available (staying in village/visiting)	13,081	64.4
Has ASHA available (staying in village/visiting)	12,492	61.1

**District-level characteristics (n** **=** **574)**	**Median, IQR**	

Percentage of women with primary education	36.1, 24.0	
Percentage of fourth or higher birth order children	18.4, 19.5	
Percentage of villages with subcentre	37.7, 24.1	
Percentage of households in the highest wealth quintile	14.7, 18.4	

Note: ANM = auxiliary nurse midwife; ASHA = accredited social health activist; PHC = primary health centre.

**Table 2:** DPT coverage rates (95% confidence intervals) for 6- to 23-month-old infants in villages across India, by individual- and household-level characteristics.

Characteristics	Coverage rate (95% confidence interval^a^)		
	DPT1^b^	DPT3|DPT1^c^	DPT3^d^
**Total**	81.4 (80.9, 81.8)	78.9 (78.5, 79.3)	62.9 (62.3, 63.4)

**Sex, birth order**			
Boy, birth order ⩽3	85.4 (85, 85.9)	81.3 (80.7, 81.9)	68.5 (67.8, 69.1)
Girl, birth order ⩽3	85 (84.5, 85.5)	80.6 (80, 81.2)	67.5 (66.7, 68.2)
Boy, birth order ⩾4	71.4 (70.3, 72.4)	71.6 (70.4, 72.9)	49 (47.8, 50.2)
Girl, birth order ⩾4	66.4 (65.3, 67.6)	68.6 (67.2, 70)	43.2 (41.9, 44.5)

**Mother’s education**			
0 years of schooling	71.5 (70.9, 72.2)	71.1 (70.4, 71.8)	48.9 (48.1, 49.6)
Years of schooling: 1–5	84.2 (83.4, 84.9)	79.3 (78.4, 80.2)	65.5 (64.5, 66.6)
Years of schooling: 6–9	91.4 (90.9, 91.9)	84.4 (83.8, 85.1)	76.6 (75.8, 77.4)
Years of schooling: 10 or more	96.2 (95.8, 96.5)	88.9 (88.2, 89.5)	85.2 (84.5, 86)

**Partner’s education**			
0 years of schooling	69.6 (68.8, 70.5)	71.4 (70.5, 72.3)	47.6 (46.6, 48.5)
Years of schooling: 1–5	79.5 (78.7, 80.3)	77.8 (76.9, 78.7)	60.3 (59.2, 61.3)
Years of schooling: 6–9	84.5 (83.9, 85.1)	80.1 (79.4, 80.8)	66.5 (65.7, 67.3)
Years of schooling: 10 or more	90.5 (90, 90.9)	83.5 (82.9, 84.2)	75 (74.3, 75.7)

**Mother’s knowledge about diarrhoea management, on scale of 0–5**			
0	75.1 (74.4, 75.8)	74.9 (74.3, 75.6)	54.4 (53.6, 55.2)
1	83.3 (82.6, 83.9)	78.7 (78, 79.4)	64.3 (63.5, 65.2)
2	88.5 (87.9, 89.1)	83.2 (82.5, 84)	72.9 (72.1, 73.8)
3–5	91.7 (90.9, 92.5)	86.3 (85.2, 87.4)	78.7 (77.4, 80)

**Mother fed colostrum during last live birth**			
No	71.4 (70.4, 72.3)	72.1 (71.1, 73.2)	49.3 (48.3, 50.4)
Yes	84 (83.6, 84.4)	80.3 (79.9, 80.8)	66.4 (65.8, 66.9)

**Mother has seen, heard, or read messages related to immunization**			
No	50.3 (48.3, 52.3)	70.8 (68.6, 73)	32.4 (30.6, 34.2)
Yes	83.5 (83.1, 83.9)	79.2 (78.8, 79.6)	64.9 (64.4, 65.5)

**Household head is Muslim**			
No	82.8 (82.4, 83.2)	79 (78.6, 79.5)	64.3 (63.7, 64.8)
Yes	71.4 (70.1, 72.8)	77.9 (76.5, 79.2)	52.9 (51.3, 54.6)

**Household head belongs to scheduled tribe**			
No	82 (81.6, 82.5)	79.9 (79.4, 80.4)	64.2 (63.6, 64.8)
Yes	78.5 (77.5, 79.5)	74.6 (73.5, 75.6)	57.4 (56.1, 58.6)

**Wealth quintile**			
Poorest	70.6 (69.7, 71.5)	69.8 (68.8, 70.8)	47.2 (46.2, 48.2)
2nd quintile	77.6 (76.9, 78.4)	75.5 (74.6, 76.3)	56.9 (56, 57.8)
3rd quintile	83.6 (82.9, 84.3)	80 (79.2, 80.7)	65.6 (64.7, 66.5)
4th quintile	90 (89.4, 90.6)	84.5 (83.8, 85.2)	75.3 (74.5, 76.2)
Richest	95 (94.5, 95.6)	88.7 (87.8, 89.5)	84 (83, 85)

a Confidence intervals are based on standard errors accounting for the sampling design.

b Percentage of infants receiving at least one DPT dose.

c Among infants who had received at least one DPT dose, the percentage receiving all three DPT doses.

d Percentage of infants receiving all three DPT doses.

**Table 3:** Multilevel logistic models for receipt of at least one DPT dose among 6- to 23-month-old infants and receipt of all three DPT doses among infants who received at least one dose of DPT, in villages across India.

Variable	Odds ratio (95% confidence interval)	
	DPT1 (n = 62,827)	DPT3|DPT1 (n = 49,806)
**Individual- and household-level**		
**Gender, birth order**		
Girl, birth order ⩽3	0.93 (0.88, 0.98)⁎⁎	0.93 (0.88, 0.98)⁎
Boy, birth order ⩾4	0.79 (0.73, 0.86)⁎⁎⁎	0.88 (0.8, 0.96)⁎⁎
Girl, birth order ⩾4	0.64 (0.59, 0.69)⁎⁎⁎	0.77 (0.7, 0.84)⁎⁎⁎

**Mother's education**		
Years of schooling: 1–5	1.24 (1.15, 1.33)⁎⁎⁎	1.16 (1.07, 1.25)⁎⁎⁎
Years of schooling: 6–9	1.64 (1.52, 1.78)⁎⁎⁎	1.34 (1.24, 1.45)⁎⁎⁎
Years of schooling: 10 or more	2.34 (2.07, 2.65)⁎⁎⁎	1.51 (1.36, 1.68)⁎⁎⁎

**Partner's education**		
Years of schooling: 1–5	1.18 (1.1, 1.26)⁎⁎⁎	1.12 (1.03, 1.21)⁎⁎
Years of schooling: 6–9	1.31 (1.23, 1.4)⁎⁎⁎	1.16 (1.08, 1.25)⁎⁎⁎
Years of schooling: 10 or more	1.47 (1.35, 1.59)⁎⁎⁎	1.12 (1.03, 1.22)⁎

**Mother's knowledge about diarrhoea management, on a scale of 0 to 5**		
1	1.19 (1.12, 1.26)⁎⁎⁎	1.04 (0.97, 1.1)
2	1.35 (1.26, 1.45)⁎⁎⁎	1.15 (1.07, 1.24)⁎⁎⁎
3–5	1.58 (1.39, 1.79)⁎⁎⁎	1.28 (1.13, 1.44)⁎⁎⁎
**Mother fed colostrum during last live birth**	1.18 (1.12, 1.25)⁎⁎⁎	1.11 (1.03, 1.18)⁎⁎
**Mother has seen, heard, or read messages related to immunization**	4.15 (3.76, 4.58)⁎⁎⁎	1.37 (1.19, 1.59)⁎⁎⁎
**Household head is Muslim**	0.56 (0.52, 0.61)⁎⁎⁎	0.75 (0.68, 0.83)⁎⁎⁎
**Household head belongs to scheduled tribe**	0.72 (0.66, 0.78)⁎⁎⁎	0.77 (0.7, 0.84)⁎⁎⁎

**Wealth quintile**		
2nd quintile	1.15 (1.08, 1.22)⁎⁎⁎	1.2 (1.12, 1.3)⁎⁎⁎
3rd quintile	1.21 (1.13, 1.31)⁎⁎⁎	1.27 (1.17, 1.38)⁎⁎⁎
4th quintile	1.42 (1.3, 1.56)⁎⁎⁎	1.5 (1.36, 1.65)⁎⁎⁎
Richest	2.01 (1.74, 2.32)⁎⁎⁎	1.96 (1.71, 2.24)⁎⁎⁎

**Village-level**		
Does not have electricity	0.8 (0.75, 0.86)⁎⁎⁎	0.89 (0.81, 0.96)⁎⁎
Has subcentre	1.05 (0.99, 1.12)	1.03 (0.96, 1.1)
Has PHC	0.94 (0.85, 1.04)	1.03 (0.93, 1.15)
Is connected by all-weather road to subcentre or PHC	1.19 (1.11, 1.28)⁎⁎⁎	1.17 (1.08, 1.27)⁎⁎⁎
Has ANM available (staying in village/visiting)	1.13 (1.06, 1.2)⁎⁎⁎	1.06 (1, 1.13)
Has ASHA available (staying in village/visiting)	1.12 (1.05, 1.19)⁎⁎⁎	1.04 (0.97, 1.12)

**District-level**		
**Percentage of women with primary education**		
Moderate [29.0, 46.5)	1.06 (0.89, 1.26)	1.08 (0.92, 1.26)
High [46.5, 94.2]	1.25 (0.95, 1.63)	1.32 (1.05, 1.66)⁎

**Percentage of youngest children of fourth or higher birth order**		
Moderate [13.1, 24.6)	0.64 (0.52, 0.79)⁎⁎⁎	0.77 (0.65, 0.92)⁎⁎
High [24.6, 53.9]	0.48 (0.37, 0.63)⁎⁎⁎	0.57 (0.46, 0.72)⁎⁎⁎

**Percentage of villages with subcentre**		
Moderate [30.2, 45.7)	1.28 (1.1, 1.49)⁎⁎	1.05 (0.92, 1.2)
High [45.7, 100.0]	1.48 (1.22, 1.79)⁎⁎⁎	1.02 (0.87, 1.21)

**Percentage of households in highest wealth quintile**		
Moderate [10.1, 22.7)	0.94 (0.8, 1.12)	1.03 (0.88, 1.2)
High [22.7, 88.3]	1.09 (0.85, 1.4)	1.06 (0.85, 1.31)
**High focus state**	0.62 (0.27, 1.4)	0.66 (0.39, 1.14)

Notes: The DPT vaccination outcomes are DPT1, receipt of at least one dose of DPT or alternatively nonvaccination with DPT; and DPT3|DPT1, receipt of all three doses among infants who received at least one dose of DPT—in other words, dropout between DPT doses 1 and 3. All regressions adjust for mother’s age and age of the child in months.

ANM = auxiliary nurse midwife; ASHA = accredited social health activist; PHC = primary health centre.

⁎ Significance: 0.01.

⁎⁎ Significance: 0.001.

⁎⁎⁎ Significance: 0.
