# Supplementary material for: Demand- and supply-side determinants of diphtheria-pertussis-tetanus nonvaccination and dropout in rural India
Source: Vaccine. 2017 Feb 15;35(7):1087–93. doi: 10.1016/j.vaccine.2016.12.024 (PMC5297340; doi:10.1016/j.vaccine.2016.12.024)
Supplement: Supplementary data 1 [file mmc1.docx]

**SUPPLEMENTARY METHODS**

**Data**

District Level Household and Facility Survey (DLHS-3), conducted during 2007–08, was designed to provide district-level estimates on important indicators of maternal and child health, family planning, and other reproductive health services. This cross-sectional survey, adopting a multistage stratified sampling design, covered 720,320 households from 601 districts in 34 Indian states and union territories. From these households, 643,944 ever-married women aged 15–49 years were interviewed.

In DLHS-3, mothers provided information on the immunization schedule of their last two surviving children born since January 1, 2004. Other information included the mother’s age and educational attainment, partner’s education, and number and sex of children ever born and surviving. Mothers were asked questions about general health knowledge and practices, such as what actions to take if a child gets diarrhoea and whether their youngest child had been fed colostrum during the first few days of life. Mothers were also asked whether they knew about (had seen, read, or heard) messages related to immunization and about the sources of information.

A household questionnaire gathered information on religion and caste of household head and ownership of various assets, among other socioeconomic and demographic variables. Household wealth index was then computed by combining household assets, amenities, and durables, and households were categorized into quintiles—from the poorest to the richest groups—based on the derived household wealth index. In the sampled villages (the primary sampling units in rural areas), information on availability of health, education, water and sanitation, electricity, and other facilities in and around the village were gathered from village-level officials—that is, the *pradhan* or the *sarpanch*—or other knowledgeable persons in the village, such as teachers. The vaccination schedule under India’s Universal Immunization Programme recommends three doses of DPT, at 6, 10 and 14 weeks. Every child is issued a vaccination card that records routine vaccinations received. In DLHS-3, detailed information—year, month, and date of the receipt of each dose—was gathered from records in vaccination cards, if the cards were shown to the enumerators. If cards were not available to be seen by the enumerator, the mother was asked about the number of DPT injections the child had received.

**Variables**

Combining data from the immunization card and maternal recall, we defined the DPT immunization status of a child. We considered three DPT vaccination outcomes: receipt of at least one dose of DPT (denoted by DPT1) versus nonvaccination with DPT; receipt of all three DPT doses (denoted by DPT3) versus non-receipt or receipt of only one or two doses of DPT vaccination; and receipt of all three DPT doses versus receipt of only one or two DPT doses among infants who received at least one dose of DPT (denoted by DPT3|DPT1).

We included individual- and household-level risk factors such as characteristics of the child, mother and the household including the child’s gender, birth order and their interaction; the mother and her partner’s years of schooling; religion and caste of the head of the household and the household wealth quintile. Mothers were tested on their knowledge of diarrhoea management based on the following five actions: give oral rehydration solution, give salt-and-sugar solution, give plenty of fluids, continue normal food, and continue breastfeeding. The yes (1) or no (0) responses were summed to get a score between 0, indicating poor knowledge of diarrhoea management, and 5, indicating adequate knowledge of diarrhoea management. We considered this score and the dummy variable indicating whether the mother during the last live birth had fed the baby colostrum during the first few days of life as proxies for mother’s general health knowledge. We also included a dummy variable indicating whether mother has seen, heard, or read immunization-related messages.

In addition to these individual- and household-level variables, we considered indicators of village-level infrastructure, such as availability of electricity, availability of subcentre and primary health centre (PHC) in the village and all-weather road connectivity with subcentre or PHC. The subcentre is the first point of contact between the primary health care system and the community and their mandate is to provide essential maternal and child health services, including immunization, to the community. The PHC serves as a cold chain point, where vaccines are stored and where the planning for immunization sessions is carried out. We therefore considered the availability of these health facilities in the village as potential factors determining vaccination coverage. We also included the availability in villages of auxiliary nurse midwives (ANMs) and accredited social health activists (ASHAs) — community health workers who help mobilize and vaccinate the rural population in India. These village-level covariates characterize the infrastructure, especially the health service delivery environment, of the village.

We also included variables summarizing the district’s demographic and socioeconomic profile and the health infrastructure. We derived district-level averages—proportion of youngest children born post January 1 2004 who are of fourth or higher birth order, proportion of ever-married women aged 15-49 years with 6 or more years of schooling, proportion of households belonging to the richest wealth quintile, and proportion of villages with a subcentre—by summing over individual-, household-, and village-level variables. The proportion of children of fourth or higher birth order is a correlate of the district-level fertility rate and the proportion of women in the district with primary education can be considered as a proxy for the educational context of the child. The district-level proportions were categorized into tertiles (low/few, moderate, and high/many) in order to allow for the possibility of nonlinear relationship with the DPT vaccination outcomes. Also, we categorized the states Bihar, Madhya Pradesh, Rajasthan, Uttar Pradesh, Jharkhand, Chhattisgarh, Uttarakhand, Orissa and Assam as ‘high-focus’ and considered this state-level dummy variable as one of the potential risk factors.

Other proximal factors correlated with DPT vaccination uptake, such as whether mother had a minimum of three antenatal care visits during last pregnancy, whether she received at least one tetanus injection and whether the child was delivered at a health facility were not considered in the search for potential determinants of different DPT vaccination outcomes. The distal risk factors, such as maternal education, household wealth quintile, and health infrastructure at the village and district levels are likely to affect the proximal factors as well and therefore are more appealing candidates for studying barriers to immunization. Inclusion of the proximal determinants as covariates in the multivariate regression may confound the relationship between the distal risk factors and DPT outcomes and were therefore not considered.

**Statistical analyses**

To model the probability of the two binary outcomes — receipt of at least one dose of DPT and receipt of all three DPT doses among infants who received at least one dose of DPT— we specified a 4-level logistic model

$${Logit P(y}_{ijkl}=1)=\beta_{0}+\beta X_{ijkl}+f_{0l}+v_{0kl}+u_{0jkl}$$

where $y_{ijkl}$, the 0/1 outcome for the *i*^th^ child in the *j*^th^ village in district *k* in state *l*, is binomially distributed. The linear predictor on the right-hand side of the equation consists of a fixed part $\beta_{0}+\beta X_{ijkl}$, estimating the coefficients for the individual-, household-, village-, district- and state-level variables; and three random intercepts attributable to states ($f_{0l}$), districts($v_{0kl}$) and villages ($u_{0jkl}$). The random intercepts are assumed to be independently and identically distributed and have variances estimated at each level - states ($\sigma_{f}^{2}$), districts ($\sigma_{v}^{2}$) and villages ($\sigma_{u}^{2}$). We exponentiated the coefficients and presented them as odds ratios for interpretation.
